# Acidic Exo-Polysaccharide Obtained from *Bacillus* sp. NRC5 Attenuates Testosterone-DMBA-Induced Prostate Cancer in Rats via Inhibition of 5 α-Reductase and Na^+^/K^+^ ATPase Activity Mechanisms

**DOI:** 10.1007/s00284-022-03098-8

**Published:** 2022-11-29

**Authors:** Abeer Y. Ibrahim, Manal G. Mahmoud, Mohsen S. Asker, Eman R. Youness, Samah A. El-Newary

**Affiliations:** 1 Department of Medicinal and Aromatic Plants Research, National Research Centre, Giza, 12622 Egypt; 2 Medical Research Division, Department of Medical Biochemistry, National Research Centre, Giza, 12622 Egypt; 3 Microbial Biotechnology Department, National Research Centre, Dokki, Giza, Egypt

## Abstract

*Bacillus *sp. NRC5 is a new strain that grows in Egyptian beaches. This strain produces acidic exo-polysaccharide that have excellent antioxidant, anti-inflammatory and anti-tumor properties. The current study aimed to introduce a new natural product feasible for prostate cancer therapies. The anti-prostate cancer of acidic exo-polysaccharide produced from marine *Bacillus *sp. NRC5 (EBPS) was determined using 7,12-dimethylbenz-(a)-anthracene; DMBA-induced prostate cancer in male Sprague Dawley rats. Rats were subcutaneously injected with testosterone (3 mg/kg/day for 3 months) and a single dose of DMBA (65 mg/kg) for induction of prostate cancer. EBPS was administrated orally at dose 200 mg/kg/day for 3 months. To study protective effect of EBPS, animals received EBPS before cancer induction, meanwhile in therapeutic effect animals received EBPS after cancer induction. EBPS debug oxidative stress and inflammatory conditions associated with prostate cancer. EBPS either protective or therapeutic material considerably reduced cancer growth rate-limiting enzyme—i.e., 5-α-reductase (46.89 ± 1.72 and 44.86 ± 2.56 µg Eq/mL) and Na^+^/K^+^ ATPase (0.44 ± 0.03 and 0.42 ± 0.02 µg Eq/mL), compared to cancer control (69.68 ± 3.46 µg Eq/mL). In addition, both cancer biomarkers—i.e., prostate-specific antigen and carcinoembryonic antigen were significantly lowered as evidence of the ability of EBPS to protect and treat prostate cancer in chemically induced rats. EBPS showed protective and therapeutic efficacy on testosterone–DMBA-induced prostate cancer rats with a good safety margin. This study may go to clinical trials after a repeated study on another type of small experimental animal, their offspring, and one big experimental animal.

**Supplementary Information:**

The online version contains supplementary material available at 10.1007/s00284-022-03098-8.

## Introduction

Prostate cancer is described as an uncontrolled growth of cells in the prostate gland. Prostate cancer develops at 50 and peaks at 60–70 years. Prostate cancer grows slower than other cancers, and cell changes need 30 years before a tumor becomes large enough to produce symptoms. Ultimately, cancer cells invade other tissues everywhere in the body. Therefore, when the symptoms appear, cancer is considered already advanced [1].

Prostate cancer symptoms include trouble passing urine, frequent urge to pass urine, particularly at night, difficult urination, weak, dribbling or interrupted flow of urine, painful or burning urination, difficult erection, decreased ejaculation fluid, painful ejaculation, bloody urine or semen, pain in the rectum, pain or stiffness in the lower back, hips, pelvis, or thighs. Prostate cancer spreads into the lymph nodes of the pelvis or throughout the body. It tends to invade the bones. So, bone pain, especially in the back, can be a symptom of advanced prostate cancer [2].

The highest presence of prostate cancer is in the USA and Canada, European countries, and then Asian countries. Meanwhile, the lowest incidence is in the Arabic people. For example, the incidence of prostate cancer was reported to be about 100–127/100,000 men in the USA, while only 3.1, 3.3, and 6.5/100,000 men in Saudi Arabia, Oman, and Kuwait [3].

Cancer is the Growing Monster in Egypt. In 2013, 115.7/100,000 males and 110.3/100,000 females were diagnosed with cancer. Moreover, the number of cases will elevate to 341,169 because of population growth of 160% and the aging population in 2050 [4].

Many tests can be used to collect information about the prostate tract, including (i) digital rectum inspection (DRE), (ii) cystoscopy, (iii) ultrasound, (iv) prostate biopsy, and (v) biological diagnosis (acid phosphate and prostate-specific antigen PSA) [5].

PSA protein is naturally produced in the prostate cells and occasionally it is penetrated the blood and can be quantified. In prostate cancer, PSA levels in the blood are higher than normal. Therefore, PSA tests are often used to follow men after prostate cancer treatment to check for signs of cancer recurrence [2]. The total PSA consist of bounded PSA and free. Free PSA is accompanied by benign prostate. Meanwhile, bound PSA is accompanied by prostate cancer. The high percentage of free PSA proposes benign rather than cancer, but cancer is more likely with a low percentage of free PSA [2].

Several prostate cancer remediations are used, including high-intensity focused ultrasound (HIFU), healthy food and lifestyle modifications, exercising, participating in support groups, and yoga [6]. Also, Hormone therapy, including abiraterone (Zytiga), enzalutamide (Xtandi), and apalutamide (Erleada), has been approved. Additionally, 5-alpha reductase inhibitors suppress the activity of 5-alpha reductase, such as finasteride (Proscar) and dutasteride (Avodart). Chemotherapy drugs, such as docetaxel (Taxotere) and cabazitaxel (Jevtana), can help men live longer. Immunotherapy increases the body’s immune system to fight off or destroy cancer cells. Vaccines can help treat, not prevent, prostate cancer as sipuleucel-T (Provenge). However, these drugs are costly, reducing their availability in a developing country, like Egypt. Therefore, it is necessary to search for Egyptian natural materials that would be more available and more acceptable.

Our previous studies discovered several materials from the Egyptian environment with promising biological values as microbial polysaccharides and plant extracts. Many polysaccharides produced from microorganisms in Egypt exhibited an ability to treat oxidative stress and related diseases, such as cancer. For example, an acidic exo-polysaccharide produced from Marine *Bacillus amyloliquefaciens* 3MS 2017 recorded antioxidant, anti-inflammatory, and protective and therapeutic anti-tumor activities in chemically induced mammary carcinomas in rats [7, 8]. These results encouraged our team or further investigation on these natural polysaccharides. EBPS, the tested polysaccharide in the current study, is an acidic exo-polysaccharide obtained from Bacillus sp. NRC5, which is grown in the Egyptian red sea coastal region. EBPS contains several important active groups: OH, C–H, COO^−^, C–O–C, S=O, and C–O–S groups. EBPS has a low molecular weight and contains β glycosidic linkage. EBPS consists of glucose, galactose, and mannouronic acid with a molar ratio of 1.0:1.7:0.8. EBPS appeared in vitro antioxidant capacity as (i) reducing power, (ii) ROS, NOS, and free radicals scavengers, (iii) metal chelator, and (iv) lipid oxidation inhibitor. EBPS showed selective anti-inflammatory against COX-2 compared to COX-1. EBPS exhibited anti-cancer against MCF-7 and PC3 cell lines and it recorded good IC_50_ and IC_90_: 70.60 and 119.40 mg/mL on PC3 cells. EBPS at a dose of 200 mg/kg showed anti-tumor, immune-modulatory action, ability against Ehrlich Ascites Carcinoma in mice (EAC) as (i) prolongation in life span, (ii) improvement in hematological parameters, and (iii) amelioration in tumor biomarkers [9]. The same dose (200 mg/kg) of EBPS showed a therapeutic and protective effect on chemically induced mammary carcinomas through (i) decreasing cancer growth rate-limiting enzymes (aromatase and Na+/K+ATPase), (ii) modulating female sex hormones (estrogen and progesterone), (iii) alleviating oxidative stress, and (iv) suppressing COX-2 production.

The current study is an effort to find new materials from a natural origin that can treat and protect from prostate cancer and are available. The chemotherapy and chemopreventive effects of EBPS were studied in testosterone–DMBA-induced prostate cancer in male rats.

## Materials and Methods

### Production and Isolation of EBPS from *Bacillus* sp. NRC5

The isolated strain from marine was placed in the collection culture at the Microbial Biotechnology Department, National Research Center, Dokki, Giza, Egypt. The production of exo-polysaccharide (EBPS) by *Bacillus *sp. NRC5 was performed by flask fermentation using the previously reported media and assay by El-Newary et al. [7].

### Chemicals

Testosterone and 7,12 di-methylbenze-ɑ-anthracene (DMBA) were purchased from Sigma-Aldrich, USA. Kits of liver and kidney functions were obtained from Bio diagnostic, Egypt. ELISA kits for hormones determination: testosterone and Leutinizing hormone, rate cancer growth-limiting enzymes: 5-α reductase and Na^+^, K^+^ ATPase activities, carcinoembryonic antigen (CEA) and prostate-specific antigen (PSA), and anti-inflammatory biomarkers: cyclooxygenase-1 and cyclooxygenase-2 activities were purchased from Sunlong Biotech Co., LTD, Ping Shui Street, Gong Shu District, Hangzhou, Zhejiang, China, E-mail: Sales@Sunlongbiotech.Com. Antioxidant parameters kits were purchased from Bio diagnostic, Egypt. Ethylenediaminetetraacetic acid (EDTA), Sodium dihydrogen phosphate, and disodium monohydrogen phosphate were purchased from Fin Chem Ltd. All chemicals and solvents used were analytical grades.

### Determination of the In vivo Anti-prostate Cancer of EBPS from *Bacillus* sp. NRC5

#### Animals

Adult male Sprague Dawley rats (130 rats, 130–150 g weight, and three-month-old) were purchased from the animal house of the National Research Center. Animals were kept under the laboratory conditions (temperature 25–30 °C, 60–65% humidity, and the light cycle is 12/12 h). Diet was a standard diet purchased from the animal house of the National Research Center. Food and water were accessible ad libitum*.*

#### Experimental Protocol

Animals were adapted under laboratory conditions for 1 week. Animals were divided into three main groups (Supplementary Fig. 1). *The first main group* was a negative group (*n* = 10), which received saline for 6 months and was kept as a negative control. *The second main group* was the cancer control group (*n* = 30), which was subcutaneously injected with testosterone (3 mg/kg body weight dissolved in corn oil) for 3 months and then these rats were intraperitoneally injected with DMBA (65 mg/kg body weight dissolved in corn oil and saline by 1:1). Finally, these rats were kept under laboratory conditions for another 3 months.

*The third main group* was the EBPS group (*n* = 90 rats), which was divided into three subgroups:

•*The positive subgroup* orally received EBPS at 200 mg/kg body weight as a tenth of the LD_50_ [9] for 3 months (*n* = 30). Then, it was kept under laboratory conditions for another three months as a positive control for studying the sub-chronic toxicity of the tested material.
•*The therapeutic subgroup* was first subcutaneously injected with testosterone (3 mg/kg body weight) for 3 months and was intraperitoneally injected with DMBA (65 mg/kg body weight as a single dose). Finally, these rats were treated with EBPS at 200 mg/kg body weight for another 3 months (*n *= 30).
•*The protective subgroup* received EBPS at 200 mg/ kg body weight for 3 months (*n* = 30). It then was subcutaneously injected with testosterone (3 mg/kg body weight) for 3 months, followed by DMBA intraperitoneal injection (65 mg/kg body weight).

To achieve prostate cancer incidence, blood samples were obtained from the lateral tail vein of animals that chronically administrated testosterone and DMBA. Prostate-specific antigen (PSA) was determined in these samples as a specific prostate cancer biomarker detector [10]. Animals that reached more than 20-ng/mL PSA were considered prostate adenocarcinoma animals and used for the treatment [11].

After 6 months, animals were fasted overnight. Each rat was intraperitoneally injected with a mixture of ketamine and xylazine (87 and 13 mg/kg of body weight) dissolved in normal saline for anesthesia. Achieving anesthesia started 10–15 min after simultaneous injection and continued for 15–30 min [12]. Blood samples were collected from the retro-orbital plexus of anesthetic animals. Sera were obtained by centrifugation (4000×*g* and 10 min using Sigma labor zentrifugen). Organs were collected and weighed for chronic toxicity evaluation.

#### Biochemical Assessments

*Serum liver function* as total protein [13], albumin [14], and aspartate aminotransferase (AST), and alanine aminotransferase (ALT) [15] *were* spectrophotometrically determined. *Serum kidney function*, including urea [16], uric acid [17], and creatinine [18], were assayed. *In addition*, Lipid profile, including total cholesterol (TC) [19], high-density lipoprotein cholesterol (HDL-C) [20], and triglycerides (TG) [21]**,** were assayed**.** Low-density lipoprotein cholesterol (LDL-C), very low-density lipoprotein cholesterol (VLDL-C), and the risk ratio were calculated based on [20–23].

*Antioxidant Biomarkers* (1) Non-enzymatic antioxidant: GSH concentration was measured by spectrophotometry at 405 nm [24] and the unit of concentration was mg/g tissue using Ellman’s reagent (5,5ʹ-dithiobis 2-nitrobenzoic acid; DTNB), which was reduced by thiol groups to form 1 mol of 2-nitro 5-mercaptobenzoic acid/mol thiol and with maximal absorption at 412 nm. (**2**) Antioxidant enzymes were determined spectrophotometrically; Glutathione reductase (GR) activity was measured according to [25] and the amount of the enzyme reducing 1-μmol GSSG per min per mg protein was regarded as 1 activity unit. Glutathione-S-transferase (GST) activity was measured according to [26] and the amount of the enzyme that conjugate 1, chloro-2, 4-dinitrobenzene with reduced glutathione per min per mg protein was regarded as 1 activity unit. Glutathione peroxidase (GPx) activity was assayed according to [27] and the amount of the enzyme converting 1-μmol GSH per min per mg protein was taken as 1 activity unit. Catalase (CAT) activity was determined by following decomposition of H_2_O_2_ according to the method of [28]. Superoxide dismutase (SOD) was measured according to [29] as the reduction suppression rate of nitro blue tetrazolium salt and for 1 unit of activity, the amount of protein was taken which provided 50% inhibition of nitrotetrazolium reduction under standard conditions.

*Cancer Rate-limiting enzymes: 5-α reductase and ɑ1-Na, K ATPase* activities were estimated in the prostate homogenate using ELISA kits. *Testosterone and luteinizing hormone* levels at 45 and 90 days were assessed in sera samples using ELISA kits. Anti-inflammatory biomarker enzyme activities (COX-1 and COX-2) and tumor biomarkers (carcinoembryonic antigen (CEA) and PSA were determined in the sera samples using ELISA kits.

### Statistical Analysis

Data were presented as mean ± SE (*n* = 10 replicates). Comparisons among groups were performed by one-way analysis of variance ANOVA test at *P* ≤ 0.05 followed by Tukey comparison test using IBM-SPSS (version 25) followed by a post hoc test.

## Results

### Effect of EBPS Administration on Prostate Cancer Characters

Data in Table 1 show the effect of EBPS on characters of testosterone–DMBA-induced prostate cancer in male rats. All of cancer control, therapeutic, and protective groups started with 21 animals. EBPS significantly increased survival percent from 33.33% in cancer control to 71.43 and 47.62% in therapeutic and protective groups, respectively, after 3 months of administration (*P* ≤ 0.05).

**Table 1:** Effect of EBPS on characters of animals in chemically induced prostate cancer after 3 months of induction

Group parameter	–ve control	Cancer control	Therapeutic group	Protective group
Succeeded induced animal number at start	10	21*^a^	21*^a^	21
Survived animal number at end of experiment	10	7*	15*^$^	10
Survival (%)	100	33.33^*^	71.43*^$^	47.62
Body weight mean (g)	213 ± 0.98	197.37 ± 0.86*	203.88 ± 0.91*^$d^	200.0 ± 1.01*^$d^
Body weight increasing or decreasing (%)	( +) 43.66 ± 0.56	(–) 7.34 ± 0.22*	(–) 4.28 ± 0.12*^$^	(–) 6.1 ± 0.13*^$^
Prostate weight (g/ 100 g body weight)	0.232 ± 0.001	0.692 ± 0.02*	0.41 ± 0.01*^$^	0.55 ± 0.02*^$^
Tumor weight (g/100 g body weight)	0.00	0.45 ± 0.005	0.18 ± 0.004*^$^	0.275 ± 0.001*^$^
Tumor weight increase or decrease (%)	0.00	66.47 ± 0.91	43.41 ± 1.01^$^	56.28 ± 1.03*^$^
Tumor volume (mm^3^)	0.00	0.68 ± 0.005	0.39 ± 0.003^$^	0.52 ± 0.001*^$^

Data are presented as mean ± SD (*n* = 20, except -ve control, *n* = 10). Data were analyzed by one-way ANOVA followed with Tukey tests as post hoc for multiple comparisons using IBM-SPSS (version 25). *P* ≤ 0.05 was considered as significant difference

Appearance of * means significant as compared to negative control, whereas appearance of $ means significant as compared to diseased animal group. Presence of the same letter means insignificant difference between these groups

Relative tumor weight was calculated in relation to body weight, body weight increasing, or decreasing percentage were calculated in relation to –ve control at the same time, whereas –ve control was compared to animal at start time

Increase or decrease percentage of prostate were in relation to negative control

Concomitantly, body weight mean of animals administrated EBPS was significantly increased more than the cancer control (*P* ≤ 0.05). On the other hand, Prostate relative weight in cancer control animals was remarkably elevated to 0.692% ± 0.02 with increase 198.28% than the negative control (*P* ≤ 0.05). On the contrary, EBPS administration for 3 months significantly inhibited proliferation in prostate and considerably reduced prostate relative weight in therapeutic and protective groups (0.41% ± 0.01 and 0.55% ± 0.02, respectively) compared to the cancer control.

**Fig. 1:**
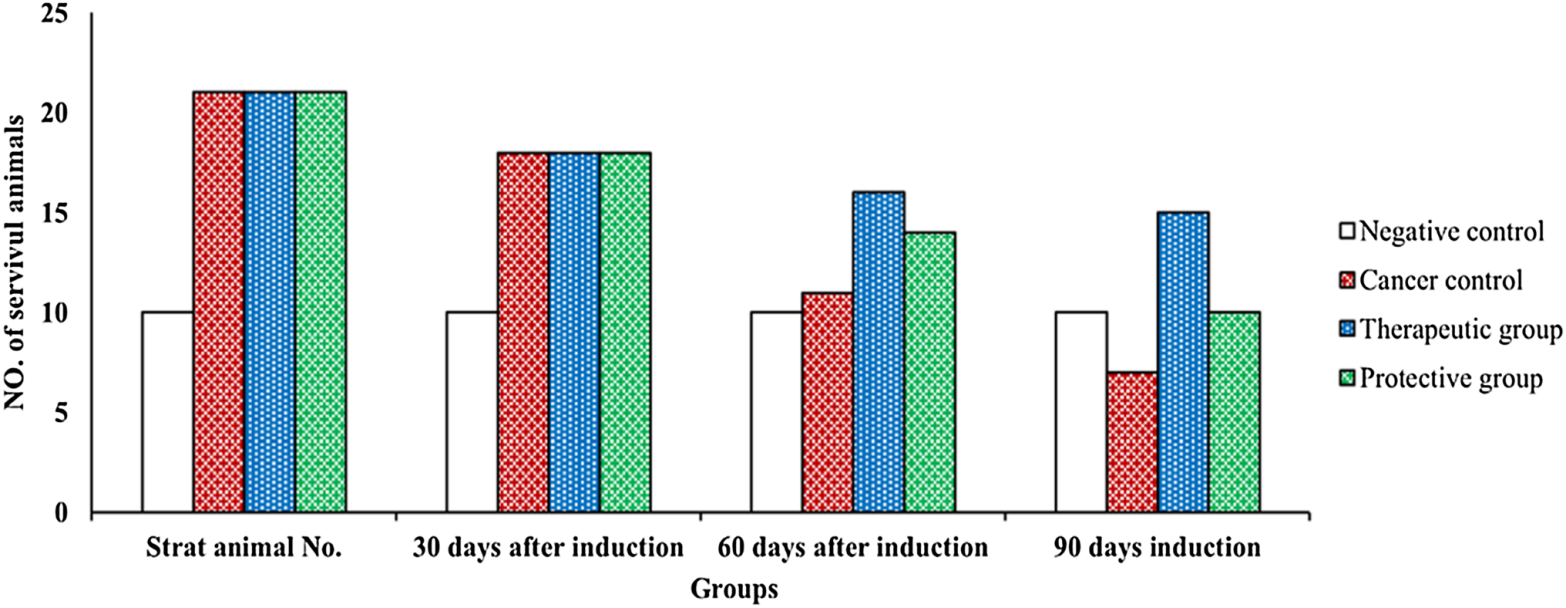
Effect of EBPS on mortality percent of animals in chemically induced prostate cancer after 3 months of induction

Cancer induction produced several tumors in the prostate of animal cancer controls with relative weight 0.45% ± 0.002 and volume 0.68 ± 0.005 cm^3^. Conversely, administration of EBPS significantly decreased prostate tumor relative weight and volume of therapeutic (0.18% ± 0.004 and 0.39 ± 0.003 cm^3^) and protective group (0.275% ± 0.001 and 0.52 ± 0.001 cm^3^), respectively, compared to cancer control (*P* ≤ 0.05). It could be evident from the explained results that the therapeutic action of EBPS was more promising than protective action on all assessed cancer parameters in Fig. 1.

### The Effect on Prostate Cancer Biomarkers

**Fig. 2:**
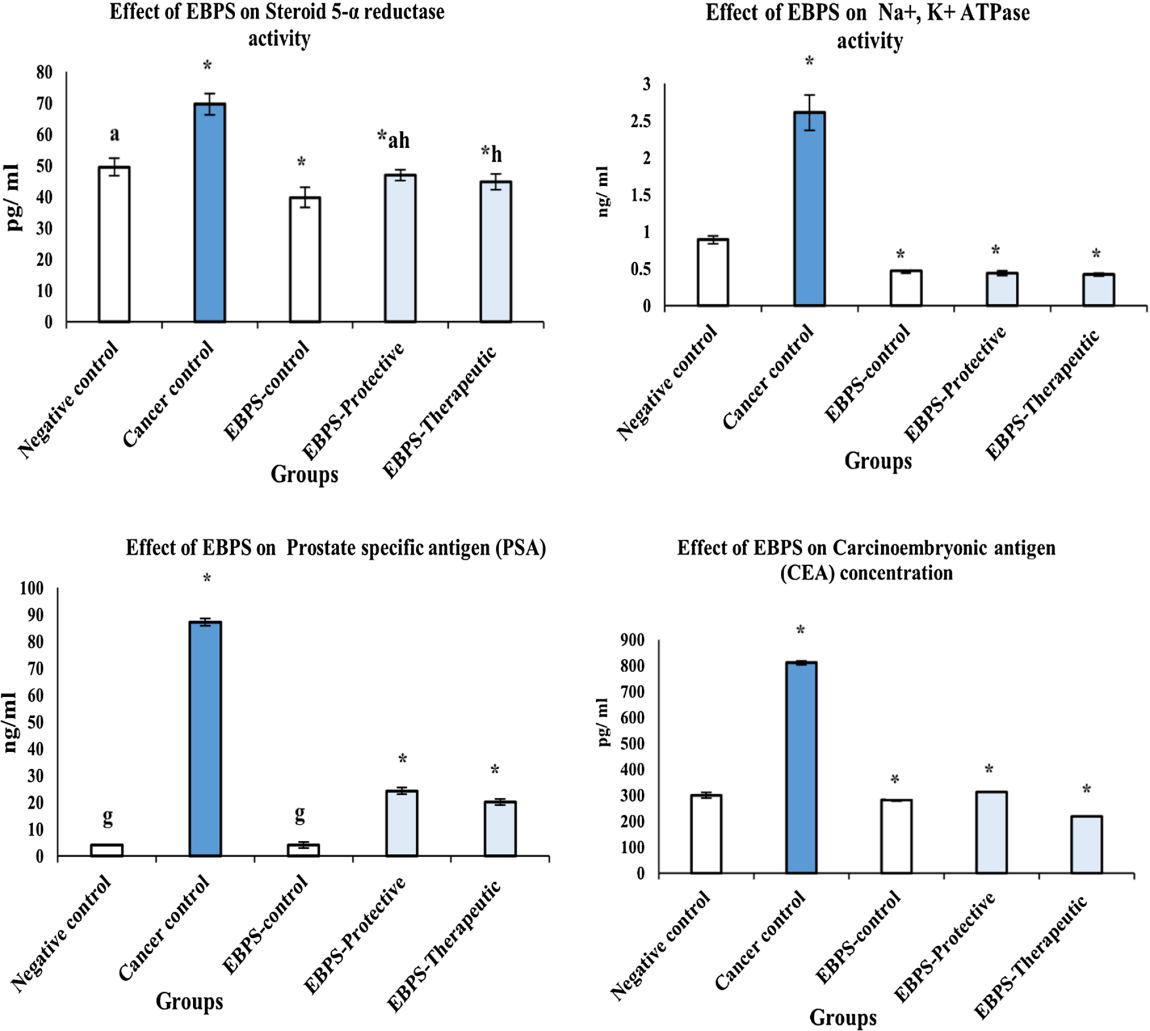
The protective and therapeutic effect of EBPS on Cancer growth rate-limiting enzymes and cancer biomarkers of DMBA-induced prostate cancer male rats. The presented data are the mean of ten replicates ± SE. ANOVA one way followed with Duncan *t* tests as post hoc for multiple comparisons. Groups having the same letter are not significantly different from each other. Treated animals were compared to the cancer control group, while the cancer group and BEPS group were compared with the negative control (*P* < 0.05). Carcinoembryonic antigen (CEA)

*Prostate-specific antigen (PSA)* of EBPS-control did not significantly change after administration of EBPS compared to the negative control (4.10 ± 0.20 and 4.11 ± 1.05 ng/mL), Fig. 2.

Significant elevation in PSA (2027%) was recorded as response to prostate cancer induction (87.23 ± 1.44 ng/mL) compared to the negative control (4.10 ± 0.20 ng/mL) (*P* ≤ 0.05). On the other hand, PSA levels of the protective and therapeutic groups significantly reduced to 24.33 ± 1.34 and 20.17 ± 1.19 ng/mL, with a 72.11 and 76.88% reduction percentage than PSA of the cancer control (*P* ≤ 0.05).

*Carcinoembryonic antigen (CEA)* exhibited significant alteration after administration of EBPS in EBPS-control compared to the negative control (281.22 ± 3.43 and 299.25 ± 11.22 pg/mL), Fig. 2.

Prostate cancer control recorded significant elevation in CEA (810.80 ± 6.67 pg/mL) that was increased more than the negative control by about 2.71 times (299.25 ± 11.22 pg/mL). On the other hand, EBPS administration significantly decreased CEA levels of the protective and therapeutic groups by about 61.43 and 73.06% than CEA of the cancer control (810.80 ± 6.67 pg/mL).

### The Effect of EBPS on Prostate Cancer Growth Rate-Limiting Enzymes

*The 5-α-reductase activity* of the EBPS-control group was significantly reduced by about 19.73% of the negative control, Fig. 2.

A significant increase was obtained in the 5-α-reductase activity of cancer control (69.68 ± 3.46 pg/mL, + 40.68%) compared to the negative control (49.53 ± 3.46 pg/mL). In contrast, a significant decrease was demonstrated in the 5-α-reductase activity of the protective and therapeutic groups in response to EBPS administration; 46.89 ± 1.72 and 44.86 ± 2.56 pg/mL with 32.75 and 35.62% than the activity of cancer control (69.68 ± 3.46 pg/mL).

*Na*^*+*^*/K*^*+*^* ATPase activity* of EBPS-control was significantly lessened to 0.47 ± 0.03 ng/mL compared to the negative control; 0.89 ± 0.05 ng/mL (*P* ≤ 0.05), Fig. 2.

Compared with the Na + /K + ATPase activity of the negative control, the Na^+^/K^+^ ATPase activity of cancer control (2.61 ± 0.24 ng/mL) was significantly raised by about 193.26%. Conversely, compared to the activity of cancer control Na^+^/K^+^ ATPase, Na^+^/K^+^ ATPase of the protective and therapeutic groups was significantly suppressed to 0.44 ± 0.03 and 0.42 ± 0.02 ng/mL.

### Effect of EBPS Administration on Antioxidant Status

**Fig. 3:**
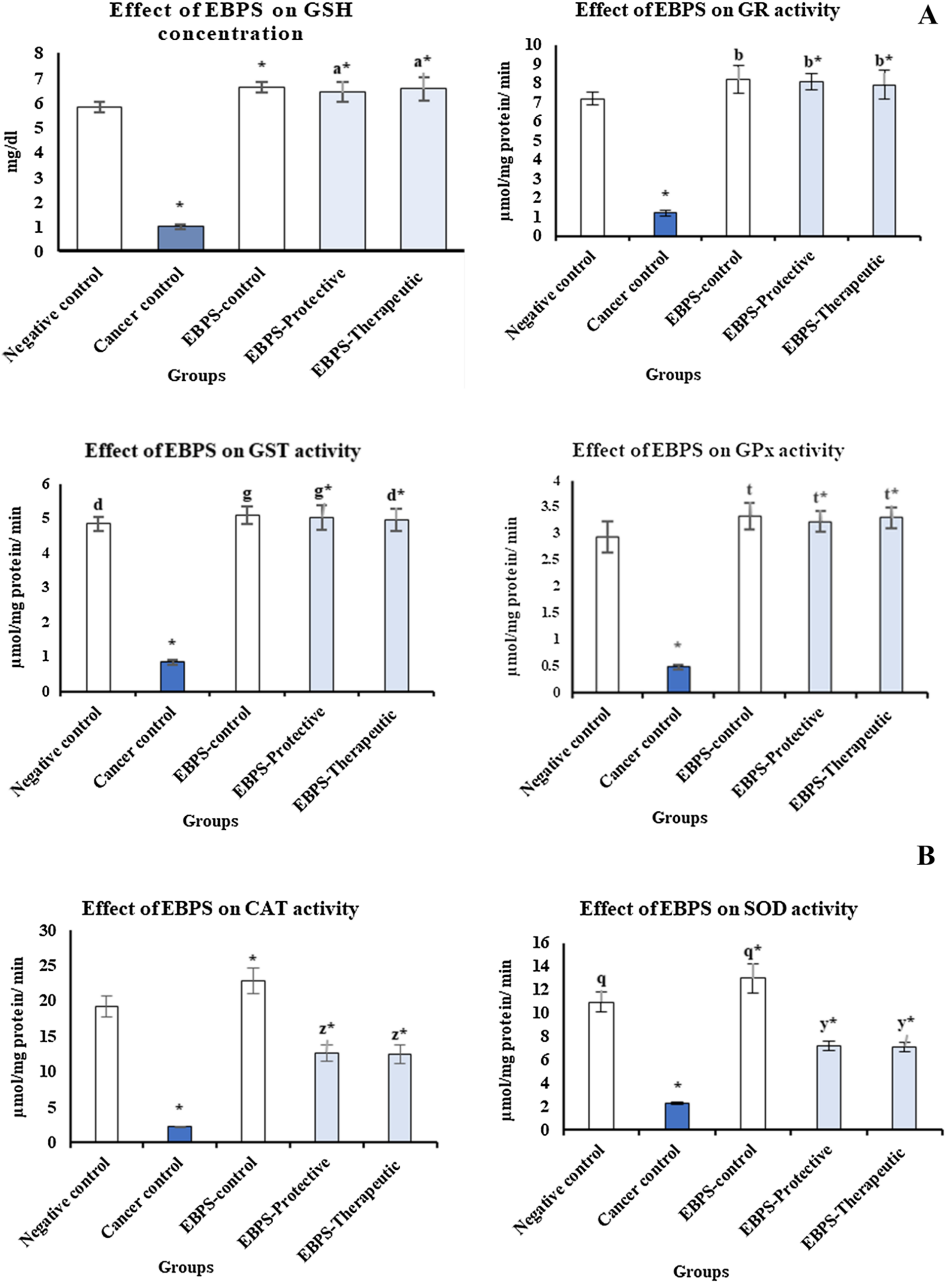
Antioxidant biomarkers of testosterone–DMBA-induced Prostate cancer in male rats treated with EBPS exo-polysaccharide, part A is GSH and its related enzymes and part 2 is SOD and CAT. The presented data are the mean of ten replicates ± SE. ANOVA one-way followed with Duncan *t* tests as post hoc for multiple comparisons. Groups having the same letter are not significantly different from each other. Treated animals were compared to the cancer control group, while the cancer group and BEPS group were compared with the negative control (*P* < 0.05). GSH is glutathione, GR is glutathione reductase, GST is glutathione-S-transferase, GPx is glutathione peroxidase, CAT is catalase, and SOD is superoxide dismutase activities

The case of oxidative stress was correlated with testosterone–DMBA administration in cancer control rats, where antioxidant biomarkers significantly decreased than the negative control. Non-enzymatic antioxidant, GSH, was significantly retarded by about 5.90 times more than the negative control. Glutathione-related enzymes, GR, GST, and GPx, were also significantly constringed to 1.22 ± 0.16, 0.85 ± 0.07, and 0.49 ± 0.04 µmol/mg protein/mint, respectively, instead of 7.19 ± 0.33, 4.82 ± 0.21, and 2.94 ± 0.29 µmol/mg protein/mint in the negative control (*P* ≤ 0.05) Fig. 3

Adversely, administration of EBPS for 3 months recorded an anti-oxidative stress effect that was represented as a significant improvement in antioxidant system of the protective and therapeutic groups compared to the cancer control. EBPS protected the antioxidant system of the protective group; GSH, GR, GST, GPx, SOD, and CAT were significantly enhanced to reach 6.42 ± 0.39 mg/dL, 8.08 ± 0.44, 5.05 ± 0.36, 3.23 ± 0.20, 12.66 ± 1.09, and 7.19 ± 0.40 U/ mg protein, respectively, compared to the cancer control (*P* ≤ 0.05).

EBPS recovered the antioxidant system, GSH, GR, GST, GPx, SOD, and CAT, of the therapeutic group, toward regular level (6.56 ± 0.46 mg/dL, 7.91 ± 0.76, 4.98 ± 0.33, 3.30 ± 0.20, 12.53 ± 1.30, and 7.12 ± 0.41 U/ mg protein, respectively), compared to cancer control (*P* ≤ 0.05).

### Effect on Inflammatory Biomarkers

**Fig. 4:**
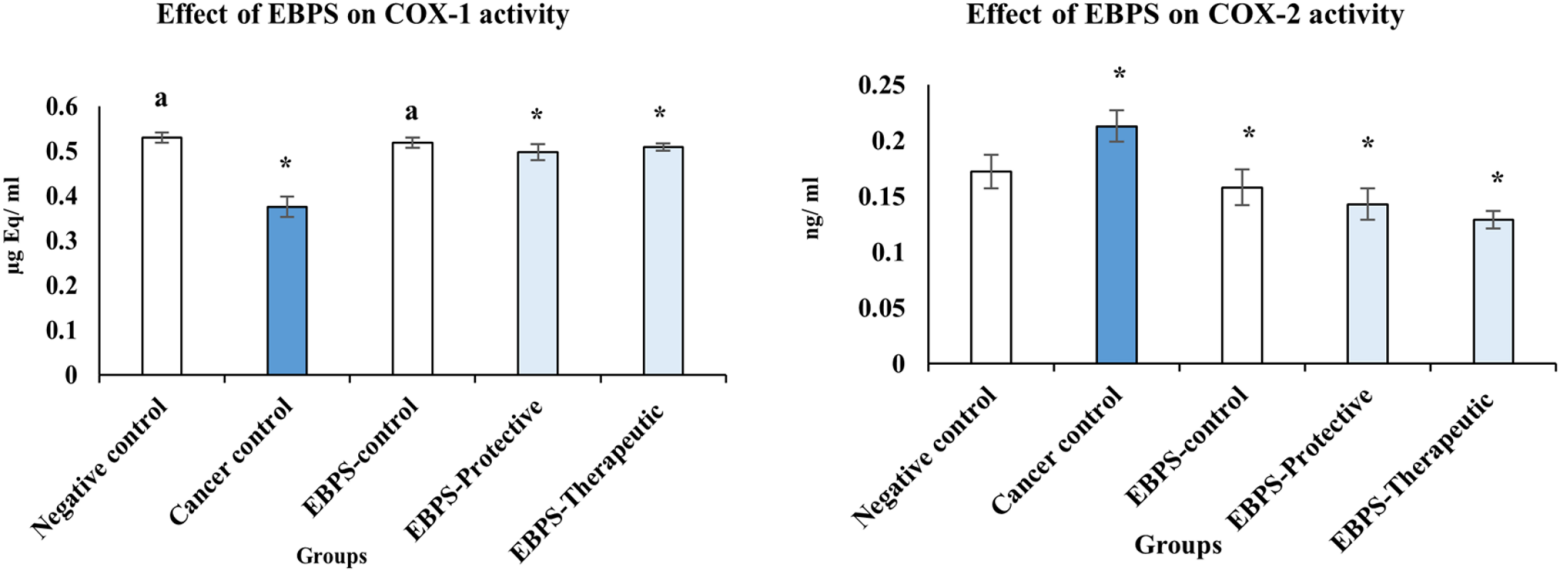
The protective and therapeutic effect of EBPS on inflammation rate-limiting enzymes of testosterone–DMBA-induced Prostate cancer in the male rat. The presented data are the mean of ten replicates ± SE. ANOVA one-way followed with Duncan *t* tests as post hoc for multiple comparisons. Groups having the same letter are not significantly different from each other. Treated animals were compared to the cancer control group, while the cancer group and BEPS group were compared with the negative control (*P* < 0.05)

Case of inflammation appeared concurrently with prostate cancer induction, where cancer group rats produced more COX-2 (0.213 ± 0.01 ng/mL with 23.84%) and less COX-1 (0.375 ± 0.02 µg Eq/mL, with 29.25%), compared to the negative control (Fig. 4).

EBPS showed a selective COX-2 inhibitory effect, not COX-1. In comparison with cancer control, EBPS caused a significant increase in COX-1 production (0.498 ± 0.02 µg Eq/mL, 32.80%) synchronous with a considerable decrease in COX-2 synthesis (0.143 ± 0.01 µg Eq/mL, 32.86%) in the protective group (*P* ≤ 0.05).

EBPS attenuated the inflammatory status recorded with prostate cancer induction in the therapeutic group and exhibited a selective anti-inflammatory effect against COX-2. As a result, the production of COX-2 was significantly reduced by about 39.44%. Meanwhile, COX-1 production was induced by approximately 35.73% more than the negative control (*P* ≤ 0.05).

There are insignificant differences between COX-1 in EBPS-control and COX-1 in the negative control. Meanwhile, EBPS-control rats produced COX-2 less than the negative control (0.158 ± 0.01 and 0.172 ± 0.02 ng/ mL, respectively) (*P* ≤ 0.05).

### Effect on Sex Hormone Production

**Fig. 5:**
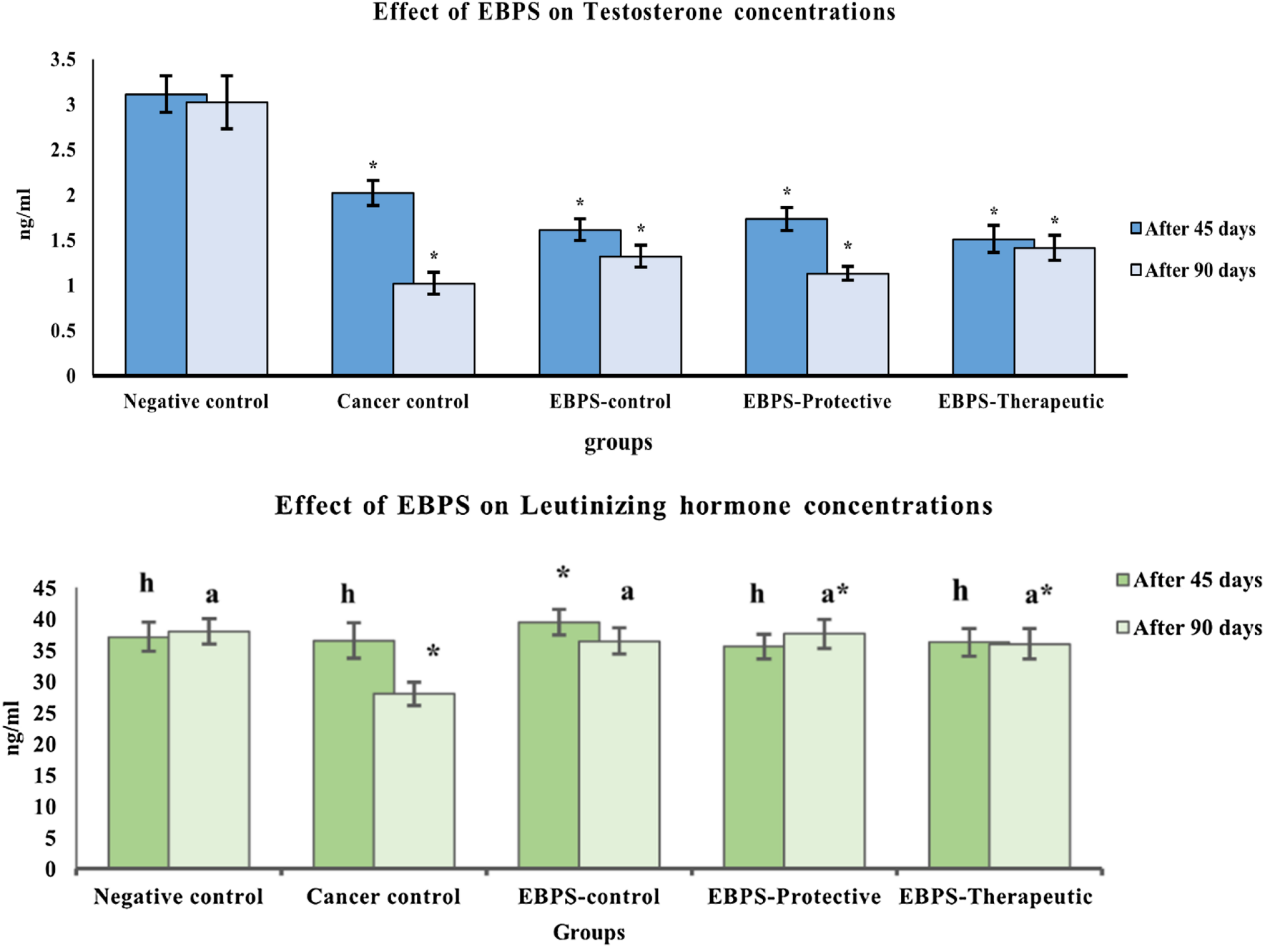
The protective and therapeutic effect of EBPS on rat sex hormones of testosterone–DMBA-induced Prostate cancer in the male rat. The presented data are the mean of ten replicates ± SE. ANOVA one-way followed with Duncan t tests as post hoc for multiple comparisons. Groups having the same letter are not significantly different from each other. Treated animals were compared to the cancer control group, while the cancer group and BEPS group were compared with the negative control (*P* < 0.05)

Administration EBPS caused a reduction in testosterone production after 45 and 90 days (1.61 ± 0.10 and 1.32 ± 0.10 ng/mL, respectively) that was significant with the negative control (3.11 ± 0.20 and 3.02 ± 0.30 ng/mL, respectively) (Fig. 5).

On the contrary, chronic administration of TS and a single dose of DMBA significantly reduced TS of the cancer group after 45 and 90 days to be 2.02 ± 0.10 and 1.02 ± 0.10 ng/mL, respectively, compared to the negative control (3.11 ± 0.20 and 3.02 ± 0.30 ng/mL, respectively). EBPS, either as a protective or therapeutic agent, reduced TS levels to 1.73 ± 0.10 and 1.51 ± 0.20 ng/mL after 45 days, which was significant compared to the cancer control (2.02 ± 0.10 ng/mL). An opposite observation was recorded after 90 days, where TS levels in the protective and therapeutic groups were significantly induced to be 1.13 ± 0.10 and 1.41 ± 0.10 ng/mL as compared to the cancer control, 1.02 ± 0.10 ng/mL.

The LH hormone of EBPS-control that was force-fed EBPS for 45 and 90 days did not change LH significantly compared to the negative control.

LH level of the cancer control did not alter significantly after 45 days. Meanwhile, after 90 days, the LH level significantly decreased to 28.00 ± 1.84 ng/mL, compared to the negative control, 38.00 ± 2.08 ng/mL. On the contrary, LH of the protective and therapeutic groups was significantly elevated to 37.67 ± 2.32 and 36.02 ± 2.43 ng/mL after 90 days compared to the cancer control (28.00 ± 1.84 ng/mL).

### Effect of EBPS Administration on the Safety Profile

To recommend the EBPS as a prostate cancer remediation, the safety profile of the EBPS was evaluated over 90 days, with mid-term sub-chronic toxicity study. In the current study, we assessed the toxicity of EBPS through its effect on the relative weight of vital organs (Supplementary Table 1 and Supplementary Fig. 2 & 3), liver and renal functions (Supplementary 2 and 3), and lipid pattern (Supplementary Table 4) of EBPS-treated rats and cancer groups by comparing them to the negative control group.

## Discussion

The present study investigated the possibility of using EBPS on testosterone-DMBA-induced prostate cancer in male rat protection and treatment. Rats with prostate cancer were characterized by a significant elevation in (i) cancer growth rate-limiting enzymes, 5-α reductase and Na^+^/K^+^ATPase activities, (ii) cancer biomarkers, prostate-specific antigen (PSA) and carcinoembryonic antigen (CEA) levels, and (iii) inflammation biomarkers, COX-2 production compared with the corresponding markers in the negative control. These changes are accompanied by a disruption in hormonal levels (testosterone TS and Luteinized hormone LH), liver and renal functions, and oxidative stress conditions.

Administration of EBPS for 90 days recorded a significant ameliorative effect in the protective or therapeutic groups. Rats administrated EBPS protection and treatment appeared to have normal hormonal status, cancer growth rate-limiting enzymes, cancer biomarkers, inflammation biomarkers, liver and renal functions, and antioxidant biomarkers. EBPS-control group showed a safe margin and appeared to have healthy performance. The Anti-prostate cancer mechanism of EBPS may be mentioned and discussed through four axes, including its antioxidant characteristics, selective anti-inflammatory effect, inhibitory action on cancer growth rate-limiting enzymes (5-α reductase and Na^+^/K^+^ATPase levels), and modulatory action on sex hormonal production.

*The first axis* is the antioxidant properties of EBPS demonstrated in this in vitro and in vivo study. The relation between reactive oxygen species (ROS) and cancer has been proven in a large research number. Excess production of ROS in normal cells is considered harmful in contrast to in cancer cells as ROS are very beneficial, where they can speed up the tumorigenesis process. Cancer, prostate cancer, is always associated with oxidative stress, disease devolving, progression, and the response to the therapy [30]. The effects of oxidative stress on the cellular proteins can produce total inactivity or altered activity in function [31]. Altered protein function modulates cell signaling [30] and can activate target genes, encouraging proliferation, survival, progression, and invasiveness.

On the other hand, cells have a cellular defense system responsible for ROS detoxification. The cellular defense system consists of enzymatic antioxidants like SOD, CAT, GR, GST, GPx, and non-enzymatic antioxidants, like GSH and possibly bilirubin and uric acid. The previously mentioned antioxidant component system reduced human prostatic intraepithelial neoplasia or prostate adenocarcinoma due to reduced protein expression [32]. From the previous explanation, it could be deduced that the anti-prostate cancer activity of EBPS demonstrated in this study may be revealed by its antioxidant characteristics. EBPS exhibited in vitro antioxidant properties as it scavenged ROS, anion, and cation free radicals and NO. Interestingly, the in vivo study showed a similar trend. EBPS exhibited in vivo antioxidant actions as significant activation of antioxidant enzyme activities (CAT, GR, GST, and GP_X_) and magnifying GSH production.

*The second axis* is the selective COX-2 inhibitory effect of EBPS that was demonstrated in previous in vitro and in vivo following assessments. Chronic inflammation has been linked to cancer, which leads to the overexpression of COX-2 [33]. The relation between the overexpression of COX-2 and human prostate cancer was confirmed [34]. COX-2 mRNA and protein synthesis are elevated in prostate cancer cells compared to normal prostate cells. Furthermore, COX-2 overexpression up-regulates Bcl-2 overexpression leading to a reduction in apoptosis of prostate cancer cells.

On the contrary, COX-1 regulates angiogenesis in endothelial cells. It also involves cell signaling and maintaining tissue homeostasis. Therefore, selective COX-2 anti-inflammatory drugs such as celecoxib encourage apoptosis of LNCaP and PC3 cells. Other studies indicate that COX-2 overexpression is involved in the angiogenesis process through hypoxia-induced COX-2 expression. That leads to the up-regulation of vascular endothelial growth factor (VEGF), the main angiogenic stimulus, down-regulated by celecoxib treatment [35]. From the previous explanation, it could be concluded that the anti-prostate cancer ability of EBPS may be attributed to being an anti-inflammatory agent demonstrated in this study, in vitro and in vivo. In in vitro assessments, EBPS showed selective COX-2 inhibitory action COX-1. In in vivo, EBPS reduced COX-2 expression by 32.86 and 39.26% in the protective and the therapeutic groups, respectively, compared to the value of cancer control. In contrast, it kept cell maintenance by remaining COX-1 at optimum.

*The third axis* was the ameliorative effect of EBPS on sex hormone production manifested in this study. The increase in the testosterone level may be due to three factors that affect TS production, including (i) 5-α reductase activity that converts TS to the active form dihydrotestosterone (DHT), leading to the consumption of TS, (ii) PSA that has an inhibitory effect against TS production, and (iii) inhibin that produces from tests and inhibits TS production [36]. Our findings agreed with [36]**,** where prostate cancer induction had a significant elevation of 5-α reductase levels in sera and PSA that may substantially reduce TS production. Meanwhile, administration of EBPS either in the protective or therapeutic groups displayed a significant decrease in PSA and 5-α reductase, leading to a considerable elevation in TS production. LH is a hormone produced from the anterior pituitary gland by gonadotropic cells in females and males. LH regulates through pulses of gonadotropin-releasing hormone, stimulating Leydig cells in the male testis to produce TS. When TS levels are low, it promotes the pituitary gland to produce LH. The feedback mechanism happens when the TS level is high and inhibits the release of GnRH and LH, respectively [37].

The relation between the TS levels and prostate cancer was confirmed in native prostatic cancer, where TS level was significantly increased in cancer experiments. In contrast, other studies reported a significant decrease in TS level, and others presented an insignificant relationship between the TS level and prostatic cancer status [38]. However, the relationship between low TS and prostatic cancer was demonstrated [39]. It was found that TS levels were significantly increased after surgical castration (as an androgen suppression therapy). Furthermore, the lower total serum TS is associated with a higher proportion of predominant Gleason pattern 4, an indicator of aggressive prostate cancer [40]. In our previous research, [41] showed the same results, where serum TS decreased in the cancer control group and significantly increased after treatment with Vitex crude extract for 90 days. Thus, according to the previous data, it could be deduced that amendment in TS and LH levels may participate in the anti-prostate cancer efficacy of EBPS.

*The fourth axis* is the inhibitory action of EBPS against the cancer growth rate-limiting enzymes; 5-alpha reductase and Na^+^/K^+^ ATPase were confirmed in this study. 5-alpha reductase enzyme (5ARE) is the enzyme that converts testosterone to a more potent form of dihydrotestosterone (DHT) in the prostatic glandular. DHT binds with the androgen receptor in the cytosol to become active. Active DHT is transported to the nucleus and works as a transcription factor for prostatic gene expression, enabling prostatic cellular function. The higher concentration of intracellular prostatic DHT and the higher affinity of DHT to bind with the androgen receptor confirm the role of 5ARE in normal and pathologic prostate physiology. The concept of increasing androgens (TS and DHT) induces prostate cancer, which is widely accepted [42], decreasing androgen, either testosterone or DHT, and inhibiting 5ARE play an essential role in prostate cancer prevention. Several studies reported occurring significant increase in 5ARE in prostate cancer. 5-AR1 was increased in high-grade cancers than BPH or low- and moderate-grade cancers [43]. *In our previous study*, [41]we recorded a significant increase in 5ARE levels in testosterone-induced prostatic cancer rats, significantly reduced after treating DMBA-induced prostate cancer animals with vitex berries crude alcoholic extract. In the current study, the administration of EBPS significantly decreased 5ARE, which may have led to its anti-prostate cancer mechanism.

Na^+^/K^+^ ATPase is a transmembrane protein complex that contains ɑ-catalytic, β-regulatory, and γ-modulatory subunits. Na^+^/K^+^ ATPase plays a critical role in maintaining cell ionic and osmotic equilibrium. Na^**+**^/K^**+**^ATPase is vital for protecting ionic balance, cellular pH, and cell volume [44]**,** essential for cell survival and several cellular functions. The prostate gland’s epithelial cells accumulate aspartate and glucose using Na^+^/K^+^ ATPase to produce and secret anion citrate related to growth and proliferation. Inhibition of Na^+^/K^+^ ATPase decreased the accumulation of aspartate resulting shortage in the citrate production, consequently inhibiting prostate epithelial cell metabolism and growth. According to the proliferative action of Na^+^/K^+^ATPase, it is strongly produced in cancer cells, like prostate cancer cells [45]. Our previous study [41] and the current research showed that testosterone-induced prostate cancer rats appeared with elevated Na/K ATPase subunit levels. Because of the essential role of Na^+^/K^+^ATPase in cell survival, proliferation, adhesion, and migration, the inhibition of Na^+^/K^+^ATPase became a therapeutic target in many studies [45]. Inhibition of Na^+^/K^+^ATPase by ouabain (cardiac glycoside drug) caused cytotoxicity to a diverse of cancer and noncancerous cells [46], through suppression of Na^+^/K^+^ATPase alpha subunit, ouabain suppressed cancer cell proliferation and migration [45], and the resistance of cancer cells to anti-cancer drugs and blocked cancer invasiveness. Na^+^/K^+^ ATPase α-1 subunit has a vital role in regulating cellular kinase function through interference with Proto-oncogene, tyrosine-protein kinase Src (c-Src), in the plasma membrane [47]. The results in the presented study show that EBPS exerted anti-prostate cancer efficacy by controlling Na^+^/K^+^ATPase level, which can be considered an essential part of its mechanism of action.

## Conclusion

The current study demonstrated that EBPS has in vivo anti-prostate cancer characteristics evident by decreasing cancer biomarkers and cancer growth rate-limiting enzymes. EBPS inhibited and treated prostate cancer in male rats via various pathways, including suppressing oxidative stress and COX-2 overexpression, 5α reductase, Na^+^/K^+^ ATPase α-subunit, and testosterone biosynthesis, which were demonstrated as cancer promoters and anti-apoptotic stimulators. Findings in the current study support amalgamation of EBPS in more progressed clinical trials considered a novel natural drug or complementary drug for treating prostate cancer.

## Supplementary Information

Below is the link to the electronic supplementary material.

Supplementary file1 (DOCX 128 kb)

Supplementary file2 (DOCX 7033 kb)

Supplementary file3 (DOCX 6965 kb)

Supplementary file4 (DOCX 31 kb)
