# Supplementary figures and images for: Acidic Exo-Polysaccharide Obtained from Bacillus sp. NRC5 Attenuates Testosterone-DMBA-Induced Prostate Cancer in Rats via Inhibition of 5 α-Reductase and Na+/K+ ATPase Activity Mechanisms
Source: Curr Microbiol. 2022 Nov 29;80(1):8. doi: 10.1007/s00284-022-03098-8 (PMC9708816; doi:10.1007/s00284-022-03098-8)

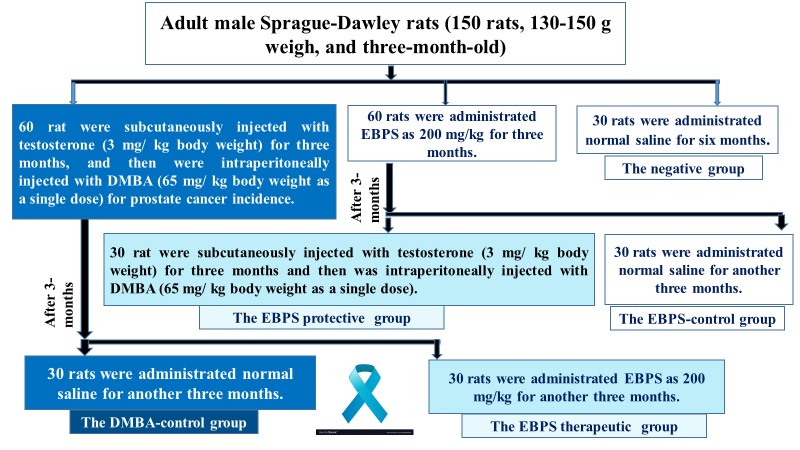


**Supplementary Figure 1.**

Supplement: Supplementary file 1 — Supplementary file1 (DOCX 128 kb) [file 284_2022_3098_MOESM1_ESM.docx]

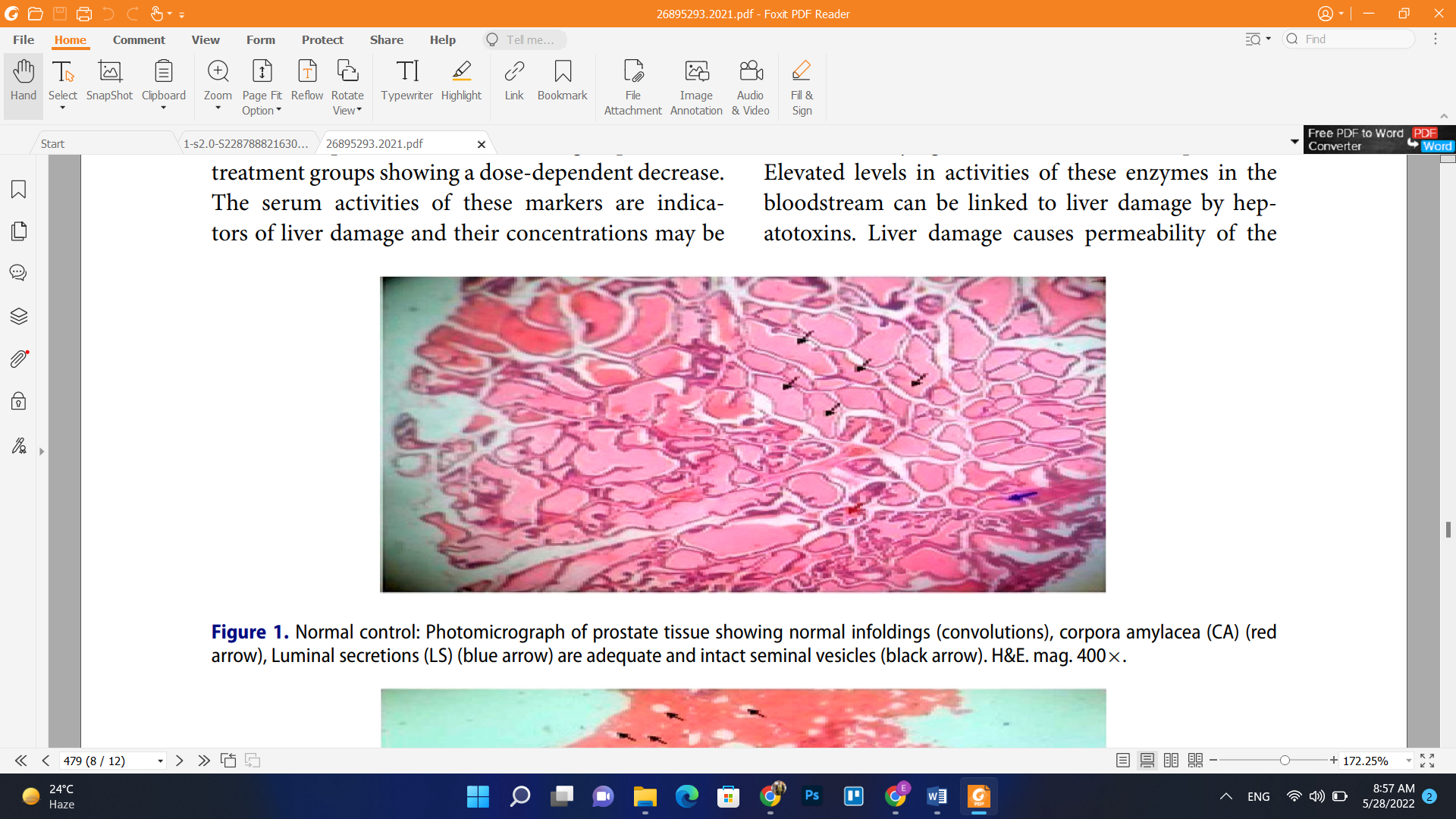


B

A

**Supplementary Figure 2.**

Supplement: Supplementary file 2 — Supplementary file2 (DOCX 7033 kb) [file 284_2022_3098_MOESM2_ESM.docx]

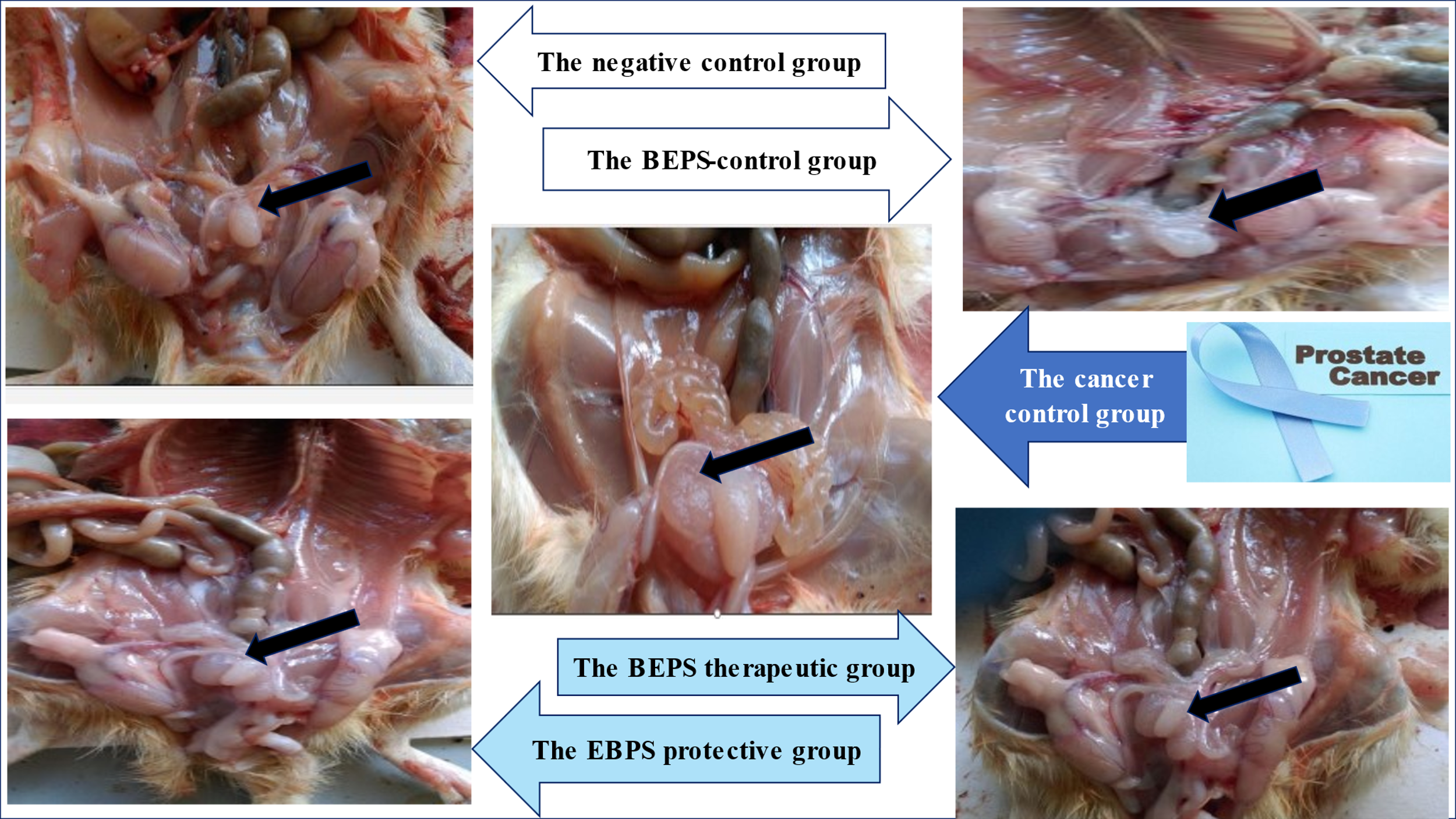


**Supplementary Figure 3.**

Supplement: Supplementary file 3 — Supplementary file3 (DOCX 6965 kb) [file 284_2022_3098_MOESM3_ESM.docx]
