# Supplementary material for: Acidic Exo-Polysaccharide Obtained from Bacillus sp. NRC5 Attenuates Testosterone-DMBA-Induced Prostate Cancer in Rats via Inhibition of 5 α-Reductase and Na+/K+ ATPase Activity Mechanisms
Source: Curr Microbiol. 2022 Nov 29;80(1):8. doi: 10.1007/s00284-022-03098-8 (PMC9708816; doi:10.1007/s00284-022-03098-8)
Supplement: Supplementary file 4 — Supplementary file4 (DOCX 31 kb) [file 284_2022_3098_MOESM4_ESM.docx]

**Acidic exo-polysaccharide obtained from** Bacillus sp. NRC5 **attenuates testosterone-DMBA-induced prostate cancer in rats via inhibition of 5 α-reductase and Na^+^/K^+^ ATPase activity mechanisms**

**Current Microbiology**

**Abeer Y. Ibrahim^1^, Manal G. Mahmoud^2^, Mohsen S. Asker^2^**

**Eman R Youness^3^ and Samah A. El-Newary^1^**

^1^Department of Medicinal and Aromatic Plants Research, National Research Centre, Giza, Egypt, 12622.

^2^Department of Medical Biochemistry, Medical Research Division, National Research Centre, Giza, Egypt, 12622.

**^3^**Department of Medical Biochemistry, Medical Research Division, National Research Centre, 12622.

Corresponding author: Samah A. El-Newary, Medicinal and Aromatic

Plants Research Department National Research Center, Giza, Egypt, 12622.

E-mail: [samahelnewary@yahoo.com](mailto:samahelnewary@yahoo.com), [samahelnewary@gmail.com](mailto:samahelnewary@gmail.com)

**ORCID iD:** Samah A El-Newary https://orcid.org/0000-0002-3141-3620

**Effect on the relative weight of vital organs**

After administering EBPS for 90 days, the relative weight of liver, kidney, lung, brain, heart, testis, and prostate of EBPS- control rats were close to the negative control. No significant differences were observed between the relative weight of EBPS-control and the negative control **(****Supplementary Table. 1, Figures 2 and 3)**. As for the relative weight of the prostate gland specifically, in EBPS-control, it did not change significantly compared to the negative control (0.233 ± 0.017 and 0.232 ± 0.013%, respectively).

Oppositely, prostate cancer induction (administration of 3 mg/ kg testosterone for 90 days and a single dose of DMBA) affected the relative weight of vital organs **(****Supplementary Table. 1)** compared to that of the negative control. Prostate cancer control appeared with inflated liver, spleen, heart, and prostate gland compared to corresponding values in the negative control (*P≤* 0.05). The relative weight of the prostate was significantly magnified by about three times that of the prostate in the negative control. In contrast, prostate cancer rats appeared with atrophied lung and brain that was significant with the values in the negative control (**Supplementary figure 3**). Both kidney and testis did not alter by polysaccharide administration when compared to those of the negative control, whereas they were shrined and enlarged, respectively, in the cancer group.

| **Parameter**  **Group** | | **Organ's weight as g/ 100 g** | | | | | | | |
| --- | --- | --- | --- | --- | --- | --- | --- | --- | --- |
|  |  | **Liver** | **Kidney** | **Spleen** | **Lung** | **Brain** | **Heart** | **Testis** | **Prostate gland** |
| **-ve control** | | **4.38±0.20** | **1.25±0.05** | **0.57±0.04 ^b^** | **0.83±0.04 ^d^** | **1.32±0.12 ^h^** | **0.50±0.05 ^k^** | **1.30±0.11 ^s^** | **0.232±0.03 ^u^** |
| **Cancer group** | | **6.81±0.30*** | **1.08±0.07*** | **0.82±0.06*** | **0.63±0.02*** | **1.00±0.03*** | **1.00±0.14*** | **1.48±0.05*** | **0.692±0.08*** |
| **EBPS** | **Control** | **4.22±0.17** | **1.23±0.03^a^** | **0.55±0.04 ^b^** | **0.84±0.04 ^d^** | **1.33±0.13 ^h^** | **0.51±0.01 ^k^** | **1.30±0.34 ^s^** | **0.233±0.01 ^u^** |
|  | **Protective** | **4.69±0.22*** | **1.21±0.02 ^a^** | **0.52±0.11*** | **0.77±0.03*** | **1.09±0.21*** | **0.53±0.10* ^q^** | **1.34±0.26* ^g^** | **0.41±0.03*** |
|  | **Therapeutic** | **4.79±0.18*** | **1.19±0.03*** | **0.59±0.11 ^b^** | **0.78±0.02*** | **1.21±0.14* ^h^** | **0.54±0.12 *^q^** | **1.32±0.41* ^g^** | **0.33±0.03*** |

**Supplementary Table. 1: Effect of EBPS exo-polysaccharide on vital organs of normal and cancer rats, chronic toxicity effect through 90day**

The presented data are the mean of 10 replicates ± SE. ANOVA one-way followed with Duncan t-tests as post hoc for multiple comparisons. Groups having the same letter are not significantly different from each other. Treated animals were compared to the cancer control group, while the cancer group and BEPS group were compared with the negative control (*P< 0.05*)*.*

EBPS- administration for three months recovered the damage that happened by prostate cancer induction. Inflated organs—i.e., liver, spleen, heart, testis, and prostate gland-significantly decreased and returned towards normalization in protective and therapeutic groups compared to cancer control. The prostate that enlarged in the cancer control was significantly reduced in the protective and therapeutic groups within normal ranges; 0.41 ± 0.03 and 0. 33 ± 0.03% with 40.75 and 52.31% reduction percent than the cancer control; 0.692 ± 0.080%. No significant difference was noticed between the relative weight of the prostate of control EBPS-treated groups and that of the negative control. It was restored close to optimal in the two treatments. Additionally, atrophied organs—i.e., lung and brain of protective and therapeutic groups were significantly increased to be within normal levels compared to cancer control.

**Effect on liver functions**

No hepatotoxic effect was recorded with chronic administration of EBPS for 90 days. Therefore, liver biomarkers—i.e., total protein, albumin, and globulin concentrations, and AST and ALT activities of the EBPS-control were close to that recorded in the negative control **(Supplementary Table. 2)**.

On the contrary, hepatotoxicity is associated with prostate cancer induction represented in significant magnification on total protein production and its two fractions—i.e., albumin and globulin compared to the negative control (*P*≤ 0.05). In addition, AST and ALT activities were significantly increased than the negative control.

**Supplementary Table 2: The protective and therapeutic effect of EBPS exo-polysaccharide on liver functions in testosterone-DMBA induced Prostate cancer in male rat**

| **Parameter**  **Group** | | **AST**  **(U/L)** | **ALT**  **(U/L)** | **AST/ ALT ratio** | **TP**  **(g/ dl)** | **Albumin**  **(g/ dl)** | **Globulin**  **(g/ dl)** | **Alb/glo**  **ratio** |
| --- | --- | --- | --- | --- | --- | --- | --- | --- |
| **Negative control** | | **65.56±4.13^a^** | **23.21±2.27 ^b^** | **2.82±0.91^d^** | **7.55±0.40 ^q^** | **4.95±0.24^w^** | **2.50±0.20 ^r^** | **1.99±0.09 ^h^** |
| **DMBA group** | | **300.25±7.97*** | **100.52±6.60*** | **2.99±1.01*** | **15.64±1.68*** | **8.30±1.0*** | **7.34±0.68*** | **1.13±0.07*** |
| **EBPS** | **Control** | **63.92±2.19 ^a^** | **22.31±1.36 ^b^** | **2.86±103 ^d^** | **7.41±1.34 ^q^** | **4.98±1.06 ^w^** | **2.47±0.08 ^r^** | **2.01±0.93 ^h^** |
|  | **Protective** | **69.63±1.37*** | **24±2.08 *^b^** | **2.90±0.83 ^g^** | **8.00±1.11* ^s^** | **5.11±1.2* ^z^** | **2.89±1.01*** | **1.77±0.07*** |
|  | **Therapeutic** | **68.16±2.11*** | **23.31±1.5* ^b^** | **2.92±0.95 ^g^** | **8.78±2.01 *^s^** | **5.13±0.9* ^z^** | **3.09±0.97*** | **1.66±0.06*** |

The presented data are the mean of 10 replicates ± SE. ANOVA one-way followed with Duncan t-tests as post hoc for multiple comparisons. Groups having the same letter are not significantly different from each other, while those having different letters are significantly different from each other. Treated animals were compared to the cancer control group while the cancer group and BEPS group were compared with the negative control *(P< 0.05*)*.*

Administration EBPS, either as a protective or a therapeutic agent, exhibited a recovering effect on liver performance. EBPS significantly ameliorated the abnormal liver biomarkers presented by cancer induction towards normalization. Therefore, liver biomarkers—i.e., total protein, albumin, and globulin concentrations, and AST and ALT activities of the protective and therapeutic groups were significantly reduced to be within the normal ranges compared to the cancer control (*P≤* 0.05). In addition, the albumin globulin ratio, which is considered an indicator of liver health, was significantly elevated to 1.77 ± 0.07 and 1.66 ± 0.06% in the protective and therapeutic groups, compared to 1.13 ± 0.07% in the cancer control.

**Effect on renal functions**

Force-feeding EBPS for 90 days had no toxic effect on the renal performance of EBPS-control compared to that of the negative control. However, insignificant differences were demonstrated between renal biomarkers—i.e., creatinine, uric acid, and urea levels of EBPS-control and negative control **(Supplementary Table. 3)**.

**Supplementary Table 3: The protective and therapeutic effect of EBPS exo-polysaccharide on Kidney functions in testosterone-DMBA induced Prostate cancer in the male rat.**

| **Parameter**  **Group** | | **Creatinine**  **(mg/dl)** | **Uric acid**  **(mg/dl)** | **Urea**  **(mg/dl)** |
| --- | --- | --- | --- | --- |
| **-ve control** | | **1.09±0.05^a^** | **5.18±0.26 ^b^** | **14.09±1.07 ^x^** |
| **Cancer group** | | **2.78±0.24*** | **8.39±0.32*** | **19.98±0.83*** |
| **EBPS** | **Control** | **1.07±0.97 ^a^** | **4.99±1.01 ^b^** | **14.06±1.31 ^x^** |
|  | **Protective** | **1.66±1.00*** | **4.49±0.94*** | **13.99±1.04 *^f^** |
|  | **Therapeutic** | **1.79±0.73*** | **4.67±1.00*** | **13.85±1.10* ^f^** |

The presented data are the mean of 10 replicates ± SD. Decreasing percentage in cancer control corresponding to ^-^ve control. Data were analyzed using ANOVA one-way followed with post hoc for multiple comparisons. The appearance of * means the significant difference between groups and –ve controls. In contrast, the appearance of letters indicates an insignificant difference between groups with the same letter. Treated animals were compared to the cancer control group while the cancer group and BEPS group were compared with the negative control (*P< 0.05).*

In counteractive, the case of renal toxicity is concurrent with prostate cancer induction, which is represented in a significant increase in renal biomarkers. Creatinine, uric acid, and urea levels compared to the negative control (*P≤* 0.05).

Meanwhile, renal biomarkers—i.e., creatinine, uric acid, and urea levels of the protective and therapeutic groups were reduced to be within optimal ranges, which was significant with those of the cancer control.

**Effect on lipid pattern**

Hypolipidemic action was associated with chronic administration of EBPS for 90 days. Lipid pattern of the EBPS-control—i.e., TG and VLDL-C, TC, and LDL-C levels were significantly diminished concerning those in the negative control. On the other hand, the HDL-C level of EBPS-control was close to the HDL-C of the negative control (**Supplementary Table. 4**).

Prostate cancer induction made a case of hyperlipidemia as a significant elevation of TG and VLDL-C (170.15%), TC (151.08%), and LDL-C (971.14%) concurrent with considerable suppression of HDL-C (25.94%) were demonstrated in the cancer control than the negative control (*P ≤* 0.05).

| **Parameter Group** | | **TG**  **(mg/ dl)** | | **VLDL-C (mg/dl)** | | **TC**  **(mg/ dl)** | | **HDL-C (mg/dl)** | | **LDL-C (mg/dl)** | | **RR** | |
| --- | --- | --- | --- | --- | --- | --- | --- | --- | --- | --- | --- | --- | --- |
| **-ve control** | | **68.97±1.44** | **13.79±0.29** | | **62.31±3.92 ^a^** | | **40.17±2.05 ^b^** | | **8.35±0.18 ^r^** | | **0.21±0.01 ^p^** | |  |
| **Cancer group** | | **186.32±4.64*** | **37.26±0.93*** | | **156.45±5.53*** | | **29.75±2.65*** | | **89.44±2.26*** | | **3.03±0.22*** | |  |
| **EBPS** | **Control** | **53.90±1.71** | **10.78±0.34** | | **57.69±1.89 ^h^** | | **39.95±1.67 ^b^** | | **8.96±0.13 ^r^** | | **0.22±0.01 ^p^** | |  |
|  | **Protective** | **61.25±2.25*** | **12.25±0.45*** | | **59.94±3.31* ^a h^** | | **36.11±1.54*^t^** | | **11.58±1.32*** | | **0.32±0.02*** | |  |
|  | **Therapeutic** | **41.38±1.75*** | **8.28±0.35*** | | **50.60±1.39*** | | **35.23±1.45* ^a^** | | **7.09±0.41*** | | **0.21±0.02* ^p^** | |  |

**Supplementary Table 4: Impact of EBPS exo-polysaccharide on lipid profile in testosterone-DMBA induced Prostate cancer in male rat**

The presented data are the mean of 10 replicates ± SE. ANOVA one-way followed with Duncan t-tests as post hoc for multiple comparisons. Groups having the same letter are not significantly different from each other. Treated animals were compared to the cancer control group while the cancer group and BEPS group were compared with the negative control (*P< 0.05).*

An amelioration of the lipid pattern of the protective and therapeutic groups was produced by EBPS administration. TG and VLDL-C, TC, and LDL-C levels were significantly reduced to the normal range. Meanwhile, HDL-C was elevated considerably to normal levels. As a result, the risk ratio significantly reduced from 3.03 ± 0.22% in the cancer control to 0.32 ± 0.02 and 0.21 ± 0.02% in the protective and therapeutic groups, respectively.
